# Supplementary material for: COVID-19 isolation and quarantine orders in Berlin-Reinickendorf (Germany): How many, how long and to whom?
Source: PLoS One. 2024 Mar 11;19(3):e0271848. doi: 10.1371/journal.pone.0271848 (PMC10927113; doi:10.1371/journal.pone.0271848)

Supporting information S2

S2 Fig. Inclusion and exclusion criteria for the data cleaning process from database export (queried) to data entries that were included in the final analysis (not duplicated).

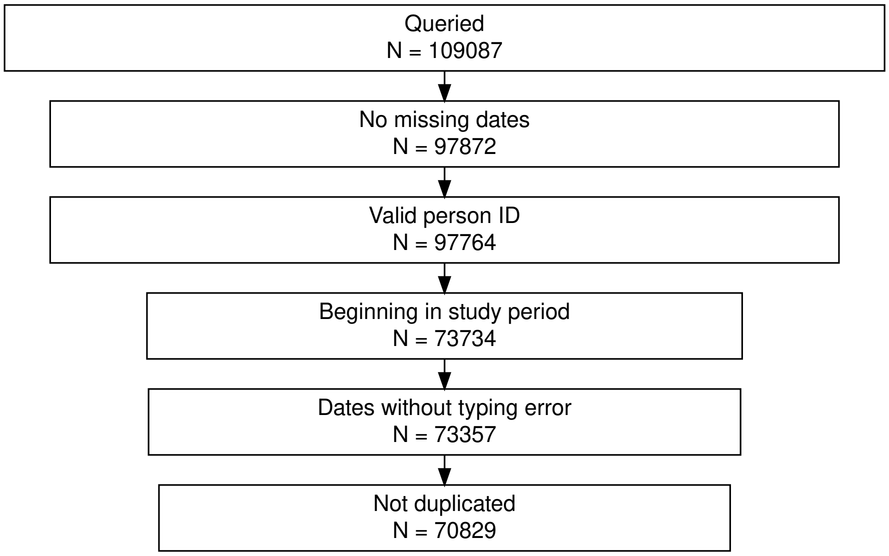

Supplement: S1 Fig — (PDF) [file pone.0271848.s002.pdf]
