# Supplementary material for: COVID-19 isolation and quarantine orders in Berlin-Reinickendorf (Germany): How many, how long and to whom?
Source: PLoS One. 2024 Mar 11;19(3):e0271848. doi: 10.1371/journal.pone.0271848 (PMC10927113; doi:10.1371/journal.pone.0271848)
Supplement: Attachment — Submitted filename: rebuttal_letter.pdf [file pone.0271848.s003.pdf]

Rebuttel letter

Points raised by Reviewer No. 1

1. Introduction on COVID-19 with emphasis on its origin and transmission (refer and cite: doi: 10.1136/postgradmedj-2020-138234).
2. Include the role of vaccination (refer and cite: doi: 10.3390/vaccines91010640)
3. Include the role of protective medications in individuals with high risk of COVID-19 (refer and cite: doi: 10.1186/s41231-021-00102-4).
4. Include a comparison of support programs in Germany with that of other regions (refer and cite: doi: 10.3389/fpubh.2022.844333)
5. Role of predictors for vaccine hesitancy in Germany. (refer and cite: doi: 10.1136/postgradmedj-2021-141365.
6. where there any inclusion and exclusion criteria among the selected populations?
7. Where there any missing records? Kindly mention in your methodology.
8. Mention if any limitations were identified in your study and also mention the follow up steps for further analysis.

Points raised by Reviewer No. 2

The authors have well versed the materials, methods and statistical analysis. The results are well described and illustrated in the figures and tables. We included the following additional citations

Answers to Reviewer No. 1

Thank you for your contributions, please find our responses to your points beneath

Answer to point 1-3 and 5

We included a short introduction to COVID-19 including its origin, pathogenesis and transmission as well as the role of vaccination, vaccine hesitancy and protective medications. We tried to keep it short to not waste the time of the reader. Please see the revised manuscript.

1. Holmes EC, Goldstein SA, Rasmussen AL, Robertson DL, Crits-Christoph A, Wertheim JO, et al. The origins of SARS-CoV-2: A critical review. Cell. 2021;184(19):4848–4856. doi:10.1016/j.cell.2021.08.017.
2. Hu B, Guo H, Zhou P, Shi ZL. Characteristics of SARS-CoV-2 and COVID-19. Nature Reviews Microbiology. 2021;19(3):141–154. doi:10.1038/s41579-020-00459-7.
3. Fiolet T, Kherabi Y, MacDonald CJ, Ghosn J, Peiffer-Smadja N. Comparing COVID-19 vaccines for their characteristics, efficacy and effectiveness against SARS-CoV-2 and variants of concern: a narrative review. Clinical Microbiology and Infection. 2022;28(2):202–221. doi:10.1016/j.cmi.2021.10.005.
4. Pires C., Global Predictors of COVID-19 Vaccine Hesitancy: A Systematic Review. Vaccines. 2022;10(8):1349. doi:10.3390/vaccines10081349.

Answer to point 4

Support programs are an important part in the response to COIVD-19. We feel that the comparison of support programs with different regions would merit its own publication. We decided to not include such a comparison to not extend the scope of our publication.

Answer to point 6 and point 7

Thank you for your point. We have not been clear enough in displaying our population, which we addressed in our revised manuscript. In our opinion we have already addressed inclusion and exclusion criteria as well as our handling of missings by stating that we took all the entries in our database (from the study period) on separation orders. Then we described which of those orders we took (all with a valid person ID with no missing data in the variables ”separation from” and ”separation until”. We then proceeded to remove all entries that we regarded as a typing error and then combining overlapping order double entries of separation orders. However we might not have been clear enough in our description so we addressed that point to make it more understandable.

Answer to point 8

Agreed, limitations are an important part in every publication. We have identified five limitations that we feel important: 1) Aim of data collection, 2) Overload of work that alters data collection 3) changes over time 4) No direct linking of cases and their contact persons and 5) indirect effects. We also wrote what we think limits the influence of these limitations. We included a paragraph on those five limitations. Also when we feel that on of our conclusions had a limitation that addressed it directly we tried to make that clear directly beside that point. We revised the section on limitations to make it more understandable and to better show their importance.

Answers to Reviewer No. 2

Thank you for you positive feedback.
