# Supplementary material for: COVID-19 isolation and quarantine orders in Berlin-Reinickendorf (Germany): How many, how long and to whom?
Source: PLoS One. 2024 Mar 11;19(3):e0271848. doi: 10.1371/journal.pone.0271848 (PMC10927113; doi:10.1371/journal.pone.0271848)
Supplement: Attachment — Submitted filename: rebuttalletter.pdf [file pone.0271848.s005.pdf]

Rebuttel letter

Points raised by Reviewer No. 3

Thank you for your thorough feedback and the time you spend on improving the paper.

1. The intro could be strengthened (the harm infliction is not a great part of the paper, is it?), and please add what the aim of the paper is.

   The introduction aims to provide a comprehensive overview, including potential negative impacts of isolation and quarantine measures. The publication is not about what adverse effects are done by quarantine and isolation but to measure the quantity of isolations and quarantines. In my eyes the aim is quite cleary covered with: *The goal of this work is to quantify and analyse isolation and quarantine orders from a local public health agency in Germany to assess differences among age groups, mean number of contacts, timeliness of quarantine orders, the number of contained or non-contained cases and to estimate the impact of the contact person tracing.* I updated the intro to improve the part about the adverse effects.

2. not sure if the analysis on the "dependence on the recommendations by the Robert Koch Institute" is that informative for the readers, I suggest to deemphasize this part throughout.

   Thank you for your input regarding the analysis on the dependence on the Robert Koch Institute's recommendations. I believe this aspect is crucial as it highlights the significance of these recommendations in guiding public health responses. I think it is important to know for future pandemics if local agencies follow or dont follow national recommendations. This analysis reflects the impact of these guidelines and shows that the national institute plays a key role. Therefore, I suggest not to deemphasize this part.

3. "delay of 4 days between contact and quarantine order": what is meant: "last contact"? what did you do with family members as there was no point contact, but usually continuous contact? (perhaps address in Methods (were they excluded for that purpose?))

   Thank you for your point regarding the 'delay of 4 days between contact and quarantine order' and the definition of 'last contact', especially in the context of family members. To clarify, I have added a paragraph in the Methods section explaining 'last contact' in the paragraph explaining the timeliness.

4. "3484 of contacts were in quarantine…": relate to the number of cases (1/18) and to the number of quarantines, and eliminate the word "of". If you are able to quantify the number of human resources (man-hours or something like that) you could relate to that measure, too.

   Point taken. I changed the sentence in the introduction to relate to the number of cases and quarantines. Unfortunately we were not able to quantify the number of human resources, although I agree that this would be a valuable addition

5. I suggest to add here the proportion of the population that received at least one isolation or quarantine order, for simplicity sake you could assume that all isolation and quarantine orders were different persons

   Thank you for your point. I added the proportion of the population as you suggested.

6. the claim that fewer days were lost due to isolation or quarantine orders is not quantified.

   I would argue that I did quantify this aspect with the sentence in the discussion: ***Compared to school closures, the intervention of isolation and quarantine orders was less costly in terms of school days lost. In Berlin, schools were closed for 124 days (from 13 March 2020 to 29 May 2020 and from 06 January 2021 to 22 February 2021). In this study, we found that the local public health agency ordered 6.8 days of separation order per schoolchild, which would be 1/18 of the number of school days lost from school closures.*** **To clarify I moved bits of this into the introduction, which is probably the better position for this sentence.**

7. I would change the conclusions. (1)the part with the Robert KochInstitute I would leave out, (2)the conclusion that the mean number of contacts is less than expected comes out of the blue as you have not said what you expected. For example: you could say that a substantial proportion of the population had been separated, that the efforts saved the children from school closures, and that a substantial number of cases occurred when in quarantine.

   As stated above I think the part with the recommendations of the RKI is important, so I would suggest to leave it in. In the paper we compared our findings to the literature that calculates around 8 contacts per day. I updated the abstract and the introduction to better clarify that point. I included your suggestions for further conclusions.

8. Methods: perhaps start with how you did the routine work and the contact tracing, how you monitored quarantined persons if they became a case (self monitoring / health department monitoring, symptom monitoring, mandatory testing?, . . .)

   Agreed, this information is missing. I added a paragraph that includes this.

9. line 73: what was the unit of observation in that database? an order for isolation, or an order to quarantine? either?

   That was indeed unclear. I clarified that I meant both.

10. what you could do and would be quite cool is to quantify the cases by the day they were removed from the public, e.g. x1 number of cases on day -2 before symptom onset (because they were in quarantine), x2 number of cases from day -1 before symptom onset, x3 number of cases on the day when they had symptom onset, etc. I think that should be feasible and would be quite good information for modelers.

    Thank you for your interesting suggestion. While this approach should indeed offer valuable insights for epidemiological modelling the information on the onset of disease is not available in the current data.

11. paragraph on "Number of quarantine orders. . . ": please give examples. Line 130: "other cases": not clear what is meant here.

    I have revised the paragraph with the aim of enhancing its readability.

12. please explain here how you dealt with household contacts from the point of view "last contact". Did you exclude household contacts because they usually had no fixed, identifiable single contact?

    Thank you for your point. I added a sentence to explain our management in household situations

13. Line 149: there are two "??", please complete manuscript

    There was a latex-problem that is hopefully solved now

14. Fig.1: Since this is a paper that gives detailed account of the efforts of a health department I suggest to overlay the number of persons that were involved with contact tracing (by week or month, if possible) to show the amount of work and resources that has gone into this effort.

    That would indeed be great if we had the numbers on that. Unfortunately we did not track the number of persons involved with contact tracing sufficiently

15. can you give also the number of cases reported, please?, perhaps also in Table 2 and or Fig 1

    Added this missing information

16. please describe point out the most important things in the figure/table (don't leave the reader alone with it)

    We previously had a discussion in the team whether to show this graph because it does not directly lead to any conclusions. In the end we felt that it has merit to give the reader a sense about the distribution of the cases and contacts over time. I added a sentence to include this information.

17. Table 2, footnotes: add what N means

    This was indeed missing

18. Line 192: the number of contacts per case is quite small. given that these include also cases in schools with likely substantially more contacts it suggests that mainly the household contacts were traced. Please add in the Limitations that this low number of contacts traced may be a reason that the percentage of contained cases of all isolation orders is not more than 14%. At any rate: could you please give a break-down of the type of contacts, e.g. household, work, school, other, or something like that? In fact, I would consider restricting the analysis of contacts to household contacts as I assume that the vast majority of contacts are household contacts. This would strengthen the paper.

    For me personally this is one of the most important findings of our study. We expect to have usually around 8 contacts per person per day (Mossong et al.) and for the contact definition we were looking for up to 14 days of potential contacts. So we should have a lot more than 2 contacts per case. Although one could argue that people cut down their contacts during the pandemic. Chinese papers reported around 10 contacts per case which is maybe why they managed to reduce the number of cases close to zero. So if we have another pandemic where we want to follow a zero-strategy we should consider stepping up the search for contacts. The other point - breaking down by type of contact - would be a valuable addition. But this information was not recorded in the software. You can see in the later parts of the publication the breakdown by age which gives an indication for the difference between schools and household. Interestingly the percentage of contained cases goes up if you have lower numbers of contacts per case - probably because you get the most important contacts only. But the absolute number of contained cases would rise the more contacts are found.

19. could you add in the table the ratio of contained cases per quarantined persons (ncc/nq) or ni/nq (isolations per quarantined persons)

    The ratio of contained cases per quarantined persons is already present as a percentage in the colum ncc-p. I added the % symbol to make it clearer. I added the ni/nq (isolations per quarantined persons).

20. I think the ratio of isolations in the 1-7 days after end of quarantine : isolations during quarantine is a useful measure that should be provided.

    I added the ratio in the column nncc/ni

21. Lines 199/200: what do you mean by “non-contained cases”? please give raw numbers here, too.

    What we mean with non-contained case is explained in the methods and in the mentioned paragraph two sentences earlier. We wanted to avoid writing *had a following isolation order during the 1 to 7 days after the quarantine order* several time.

22. perhaps provide also the percentage of the population with isolation order or quarantine order?

    We have: *The local public health agency ordered ni = 24 433 isolations and nq = 45 335 quarantines (ni-p100 = 9.2 isolation orders and nq-p100 = 17 quarantine orders per 100 inhabitants).* We hope that this gives adequate information.

    Discussion

23. Limitations are usually at the end of the paper unless the editors wish something else

    Thanks for the clarification: I moved it to the end

24. I think another limitation is that there are also cases that were never known to the health department, which is of course not the fault of the health department, however, since you do not know the true number of cases (including those never diagnosed who still contribute to transmission) you underestimate the impact of the health department

    I added the limitation.

25. Line 232: does that mean you could not calculate secondary attack rates, e.g. by type of contact (household, work, etc.) or age of contact, or an average number of cases by source case (e.g. to identify super-spreaders)?

    Yes, we would have loved to calculate secondary attack rate by type of contact, but that is not possible. Also not the other parameters. I have incorporated that information into the text.

26. Line 240: I cannot extract that or at least not easily from Table 2, please be more precise?

    I changed the sentence and included the calculations for the number: ”20 times” higher in the result section under *Analysis of quantity of isolation and quarantine orders*

27. Line 283: . . . or a reluctance of cases to give information about contacts

    Thank you for the good addition.

28. Line 295: an update has been published, however, a lot of the evidence stems from SARS and MERS; there is other publications with a focus on C-19, such as Pozo-Martin (EJE, 2023, ”Comparative effectiveness...”), Craig (JMIR 2021, ”Effectiveness of contact tracing...”) and Fetzer (PNAS, 2021, ”Measuring the scientific effectiveness...”)

    Thank you for the update. I included the Review bei Pozo-Martin.

29. Line 299: I dont understand what is meant here

    I updated the sentence, and also the corresponding part in the limitation section.

30. Line 327: “The rough estimate for the median delay of 4 days that we found must be considered as a flaw in the contact tracing. For COVID-19, an early detection of contact persons is key to hinder transmission of the disease” – please check English (particularly “flaw” and “hinder” ??)

    Changed the two sentence - hopefully for the better

31. but did adherence increase and/or administrative feasibility improve?

    Our study was not meant to measure adherence. We see that the number of non contained cases goes up the shorter the quarantine period. For measuring adherence we would needed to question cases and contacts after the order. I would have loved to do that - also to see how the agency could have improved its work. This would be something for another study.

32. Line 357: for modellers that is good information. however, again, most informative would be the proportion of cases that were isolated by day in relation to symptom onset (see above).

    I agree, but we dont have the symptom onset in our dataset.

33. The authors calculated the number of contacts per case. I think it would be more instructive to stratify by those cases who were not previously in the system as contact person and those who were. The latter will likely have very few contacts, the first might have a number of contacts. In addition it would be nice to stratify by age (of the case; very broadly, say ¡18 and 18+ year old), because children should have quite a bit more contacts than adults.

    You are perfectly right, this would be valuable information. But as stated in the limitation section we cannot directly link a contact person to a case, because this is information was not reliably stored in the software. We get a hint by the high number of contact persons within the group of children, which is very likely due to school.

Points raised by Reviewer No. 4

Thank you for your valuable feedback

1. It will help the readers understand the context better if the authors can also state in the Abstract the total number of individuals analyzed.

   Agreed. Added the figure of the total population

2. In Table 2, it will be helpful to mark the percentages as "%", and provide SD for mean values. The abbreviations are also a bit hard to understand at first glance (for example it is not intuitive to me how "ncc" refers to "quarantines that had a directly following isolation period"). As these abbreviations are used only once in Table 2, the authors can consider write them out in words or improve the labels, if possible.

   Thanks for the feedback. I Added the % and explained that "ncc" means non-contained case, which was indeed not intuitive. The table is to wide to fit the page. I dont feel that the sd for the mean values are more important than the other given values.

3. It is generally recommended to avoid using the term "elderly". I suggest changing it to "older adults", "older persons", or similar throughout the manuscript.

   Thanks for the correction. I changed it to senior citizens

Points raised by Reviewer No. 5

1. I noticed that reference 21 was not cited in the manuscript. Additionally, please renumber the references in the order they appear in the manuscript. As an example, references 36-38 currently appear in the first paragraph of the Introduction, which requires revision for proper numbering.

   Thank you for pointing that out. I changed the reference order

2. Line 11-12: "Vaccines have been developed and proven successful in preventing severe disease and death [40], ..." → I suggest change this sentence to "Vaccines have been developed and proven successful in inducing seroconversion (cite the following reference 1), preventing severe disease and death [40], ..." Reference: [1] Safety and Seroconversion of Immunotherapies against SARS-CoV-2 Infection: A Systematic Review and Meta-Analysis of Clinical Trials. Pathogens. 2021 Nov 24;10(12):1537. doi: 10.3390/pathogens10121537. PMID: 34959492; PMCID: PMC8706687.

   Added the publication from Kevin Sheng-Kai Ma, as you suggested, although I am not quite sure if it adds value.

3. Line 51-52: "Jian et al. reported on the Taiwanese digital system TRACE analysing 487 cases and 8051 contact persons [12]." → To emphasize the public health implications of these particular systems, I suggest change this sentence to: "Jian et al. reported on the Taiwanese digital system TRACE, analyzing 487 cases and 8051 contact persons [12]. Public health implications of these systems included surveillance of travel history (cite the following reference 1) and ensuring an adequate quantity of personal protective equipment (cite the following reference 2)." References: [1] Integrating travel history via big data analytics under universal healthcare framework for disease control and prevention in the COVID-19 pandemic. J Clin Epidemiol. 2021 Feb;130:147-148. doi: 10.1016/j.jclinepi.2020.08.016 [2] Big Data-driven personal protective equipment stockpiling framework under Universal Healthcare for Disease Control and Prevention in the COVID-19 Era. Int J Surg. 2020 Jul;79:290-291. doi: 10.1016/j.ijsu.2020.05.091

   Added the two publications from Kevin Sheng-Kai Ma. But as above I dont see an added value.
